# Supplementary material for: KSHV-encoded vCyclin can modulate HIF1α levels to promote DNA replication in hypoxia
Source: eLife. 2021 Jul 19;10:e57436. doi: 10.7554/eLife.57436 (PMC8315796; doi:10.7554/eLife.57436)
Supplement: Supplementary file 1. [file elife-57436-supp1.docx]

Supplementary File 1: List of primers used to amplify various HREs within vCyclin promoter and for the real time PCR.

| LTd FL F | CCGCTCGAGATAGAGTGGCGAGCGTATG |
| --- | --- |
| LTd FL R | CGACGCGTCATTAACCCAGCCAGGGGA |
| Deletion HRE1 F | CATTAACCCAGCCAGGGGAAAACCAGGG |
| Deletion HRE1 R | GTGGAGAGTCCCTGGTTTTCCCCTGGCT |
| Deletion HRE2 F | ACGGACAACGGCTCTCGCGGCCCAGCGT |
| Deletion HRE2 R | TGGGCCGCGAGAGCCGTTGTCCGTGTGT |
| Deletion HRE3 F | GTTCTCCCATCGGGGCCGCGAGAGTACG |
| Deletion HRE3 R | CTCTCGCGGCCCCGATGGGAGAACCTGA |
| Deletion HRE4 F | CTCCAGGCTCTACCACATACGCTCGCCA |
| Deletion HRE4 R | ATAGAGTGGCGAGCGTATGTGGTAGAGC |
| PDK1 F | GGATCAGAAACCGACACAAT |
| PDK1 R | ACATTCTGGCTGGTGACAGG |
| PDHA1 F | GTGATGGTCAGGAAGCTTGC |
| PDHA1 R | GCCCCGGGTGAAAGTAAAGC |
| LDHA F | GACCTACGTGGCTTGGAGA |
| LDHA R | TCCATACAGGCACACTGGAA |
