# Supplementary material for: KSHV-encoded vCyclin can modulate HIF1α levels to promote DNA replication in hypoxia
Source: eLife. 2021 Jul 19;10:e57436. doi: 10.7554/eLife.57436 (PMC8315796; doi:10.7554/eLife.57436)
Supplement: Supplementary file 2. [file elife-57436-supp2.docx]

Supplementary File 2: HIF1α binding sites on the KSHV genome in BC3 cells grown under hypoxic conditions.

| BC3_Hypoxia |  |  |  |  |
| --- | --- | --- | --- | --- |
| Region | Center of peak | Length | Peak shape score | P-value |
| 959..1035 | 994 | 77 | 1.79 | 0.04 |
| 2420..2494 | 2451 | 75 | 1.58 | 0.06 |
| 4677..4748 | 4709 | 72 | 1.8 | 0.04 |
| 5301..5377 | 5336 | 77 | 2.96 | 1.54E-03 |
| 17087..17177 | 17121 | 91 | 1.69 | 0.05 |
| 21207..21290 | 21256 | 84 | 1.83 | 0.03 |
| 22623..22698 | 22658 | 76 | 2.71 | 3.34E-03 |
| 23457..23531 | 23492 | 75 | 4.02 | 2.92E-05 |
| 23887..23958 | 23919 | 72 | 1.5 | 0.07 |
| 25725..25798 | 25760 | 74 | 2.93 | 1.69E-03 |
| 26178..26249 | 26213 | 72 | 1.71 | 0.04 |
| 28579..28651 | 28617 | 73 | 1.49 | 0.07 |
| 29563..29623 | 29587 | 61 | 1.5 | 0.07 |
| 30805..30876 | 30840 | 72 | 2.31 | 0.01 |
| 43098..43175 | 43133 | 78 | 1.58 | 0.06 |
| 58896..58973 | 58931 | 78 | 4.63 | 1.79E-06 |
| 59734..59810 | 59769 | 77 | 2 | 0.02 |
| 62542..62623 | 62595 | 82 | 1.34 | 0.09 |
| 68293..68392 | 68358 | 100 | 2.21 | 0.01 |
| 85612..85681 | 85644 | 70 | 1.94 | 0.03 |
| 90907..90982 | 90942 | 76 | 2.84 | 2.28E-03 |
| 117498..117570 | 117529 | 73 | 2.02 | 0.02 |
| 117913..118004 | 117970 | 92 | 1.63 | 0.05 |
| 119636..119707 | 119668 | 72 | 3.83 | 6.46E-05 |
| 122941..123035 | 123009 | 95 | 1.4 | 0.08 |
| 124184..124253 | 124219 | 70 | 1.76 | 0.04 |
| 124838..124913 | 124879 | 76 | 2.05 | 0.02 |
| 126217..126314 | 126280 | 98 | 5.68 | 6.86E-09 |
| 126420..126472 | 126438 | 53 | 1.73 | 0.04 |
| 129844..129914 | 129879 | 71 | 2.26 | 0.01 |
| 135251..135331 | 135299 | 81 | 2.8 | 2.59E-03 |
| 135455..135536 | 135502 | 82 | 2.01 | 0.02 |
| 135895..135982 | 135958 | 88 | 1.7 | 0.04 |
