# Supplementary material for: KSHV-encoded vCyclin can modulate HIF1α levels to promote DNA replication in hypoxia
Source: eLife. 2021 Jul 19;10:e57436. doi: 10.7554/eLife.57436 (PMC8315796; doi:10.7554/eLife.57436)
Supplement: Supplementary file 3. [file elife-57436-supp3.docx]

Supplementary File 3: HIF1α binding sites on the KSHV genome in BC3 cells grown under normoxic conditions.

| BC3_Normoxia |  |  |  |  |
| --- | --- | --- | --- | --- |
| Region | Center of peak | Length | Peak shape score | P-value |
| 5324..5382 | 5365 | 59 | 1.63 | 0.05 |
| 23478..23539 | 23514 | 62 | 2.63 | 4.21E-03 |
| 30831..30891 | 30848 | 61 | 1.31 | 0.09 |
| 58919..58981 | 58957 | 63 | 3.23 | 6.18E-04 |
| 68346..68404 | 68384 | 59 | 1.52 | 0.06 |
| 85627..85686 | 85664 | 60 | 1.42 | 0.08 |
| 90928..90985 | 90966 | 58 | 1.83 | 0.03 |
| 119656..119716 | 119692 | 61 | 2.94 | 1.65E-03 |
| 126259..126318 | 126293 | 60 | 2.39 | 8.40E-03 |
| 129860..129933 | 129902 | 74 | 1.63 | 0.05 |
| 135270..135337 | 135312 | 68 | 1.61 | 0.05 |
| 136381..136435 | 136416 | 55 | 1.48 | 0.07 |
