# Supplementary material for: KSHV-encoded vCyclin can modulate HIF1α levels to promote DNA replication in hypoxia
Source: eLife. 2021 Jul 19;10:e57436. doi: 10.7554/eLife.57436 (PMC8315796; doi:10.7554/eLife.57436)
Supplement: Supplementary file 4. [file elife-57436-supp4.docx]

Supplementary File 4: HIF1α binding sites on the KSHV genome in infected PBMCs grown under hypoxic conditions.

| PBMCs_Hypoxia |  |  |  |  |
| --- | --- | --- | --- | --- |
| Region | Center of peak | Length | Peak shape score | P-value |
| 179..309 | 224 | 131 | 2.04 | 0.02 |
| 963..1080 | 1008 | 118 | 2.74 | 3.04E-03 |
| 1355..1478 | 1434 | 124 | 1.3 | 0.1 |
| 1567..1658 | 1601 | 92 | 1.45 | 0.07 |
| 2434..2509 | 2489 | 76 | 2.28 | 0.01 |
| 5294..5424 | 5335 | 131 | 2.03 | 0.02 |
| 5460..5593 | 5504 | 134 | 1.52 | 0.06 |
| 6145..6269 | 6187 | 125 | 1.3 | 0.1 |
| 12635..12734 | 12680 | 100 | 1.29 | 0.1 |
| 13210..13322 | 13283 | 113 | 1.68 | 0.05 |
| 17079..17203 | 17114 | 125 | 1.32 | 0.09 |
| 21215..21325 | 21260 | 111 | 2.72 | 3.23E-03 |
| 22644..22746 | 22689 | 103 | 2.74 | 3.10E-03 |
| 23457..23575 | 23492 | 119 | 2.74 | 3.11E-03 |
| 23900..23965 | 23916 | 66 | 1.95 | 0.03 |
| 24771..24884 | 24798 | 114 | 2.68 | 3.64E-03 |
| 25726..25838 | 25754 | 113 | 2.82 | 2.40E-03 |
| 26191..26294 | 26235 | 104 | 2.08 | 0.02 |
| 28421..28511 | 28466 | 91 | 1.31 | 0.09 |
| 28580..28701 | 28657 | 122 | 1.49 | 0.07 |
| 30830..30924 | 30875 | 95 | 1.99 | 0.02 |
| 36599..36698 | 36644 | 100 | 1.53 | 0.06 |
| 40994..41096 | 41052 | 103 | 1.46 | 0.07 |
| 42176..42299 | 42262 | 124 | 2.07 | 0.02 |
| 48185..48277 | 48230 | 93 | 1.75 | 0.04 |
| 50722..50811 | 50767 | 90 | 1.44 | 0.07 |
| 54193..54282 | 54238 | 90 | 1.88 | 0.03 |
| 58898..59025 | 58936 | 128 | 3.92 | 4.46E-05 |
| 59753..59864 | 59797 | 112 | 1.84 | 0.03 |
| 62552..62669 | 62594 | 118 | 3.27 | 5.34E-04 |
| 68319..68448 | 68360 | 130 | 3.31 | 4.61E-04 |
| 71967..72091 | 72047 | 125 | 1.4 | 0.08 |
| 75501..75610 | 75546 | 110 | 1.33 | 0.09 |
| 77413..77544 | 77455 | 132 | 1.35 | 0.09 |
| 80051..80142 | 80096 | 92 | 1.5 | 0.07 |
| 81856..81984 | 81899 | 129 | 1.41 | 0.08 |
| 83850..83980 | 83936 | 131 | 1.28 | 0.1 |
| 85504..85601 | 85549 | 98 | 2.96 | 1.54E-03 |
| 86158..86261 | 86217 | 104 | 2.11 | 0.02 |
| 89427..89523 | 89467 | 97 | 1.51 | 0.07 |
| 90912..91032 | 91001 | 121 | 1.85 | 0.03 |
| 93810..93942 | 93855 | 133 | 2.16 | 0.02 |
| 106497..106596 | 106542 | 100 | 1.3 | 0.1 |
| 115593..115680 | 115630 | 88 | 1.41 | 0.08 |
| 117487..117621 | 117532 | 135 | 1.75 | 0.04 |
| 117928..118038 | 117994 | 111 | 1.88 | 0.03 |
| 119643..119747 | 119688 | 105 | 3.09 | 9.85E-04 |
| 122975..123068 | 123020 | 94 | 1.78 | 0.04 |
| 123665..123760 | 123692 | 96 | 1.34 | 0.09 |
| 124188..124321 | 124233 | 134 | 1.96 | 0.03 |
| 124857..124985 | 124941 | 129 | 2.66 | 3.96E-03 |
| 125278..125384 | 125353 | 107 | 1.59 | 0.06 |
| 125447..125550 | 125506 | 104 | 3.6 | 1.57E-04 |
| 126247..126350 | 126288 | 104 | 4.04 | 2.71E-05 |
| 126415..126532 | 126460 | 118 | 2.34 | 9.67E-03 |
| 129385..129502 | 129469 | 118 | 1.46 | 0.07 |
| 129842..129930 | 129887 | 89 | 2.81 | 2.48E-03 |
| 131178..131288 | 131219 | 111 | 1.34 | 0.09 |
| 135240..135369 | 135285 | 130 | 3.03 | 1.21E-03 |
| 135488..135577 | 135533 | 90 | 2.41 | 7.97E-03 |
| 135655..135771 | 135727 | 117 | 1.49 | 0.07 |
| 135923..136016 | 135968 | 94 | 2.68 | 3.70E-03 |
| 136346..136435 | 136390 | 90 | 3 | 1.36E-03 |
| 136574..136668 | 136624 | 95 | 2.21 | 0.01 |
| 136741..136839 | 136784 | 99 | 1.39 | 0.08 |
| 136908..137018 | 136953 | 111 | 1.81 | 0.04 |
| 137189..137268 | 137235 | 80 | 2.43 | 7.49E-03 |
| 137307..137438 | 137349 | 132 | 3.42 | 3.11E-04 |
| 137809..137866 | 137833 | 58 | 1.44 | 0.07 |
