# Supplementary material for: KSHV-encoded vCyclin can modulate HIF1α levels to promote DNA replication in hypoxia
Source: eLife. 2021 Jul 19;10:e57436. doi: 10.7554/eLife.57436 (PMC8315796; doi:10.7554/eLife.57436)
Supplement: Supplementary file 5. [file elife-57436-supp5.docx]

Supplementary File 5: HIF1α binding sites on the KSHV genome in infected PBMCs grown under normoxic conditions.

| PBMCs_Normoxia |  |  |  |  | |
| --- | --- | --- | --- | --- | --- |
| Region | Center of peak | Length | Peak shape score | | P-value |
| 1078..2263 | 1420 | 1186 | 4.253579 | 1.05E-05 | |
| 4197..5492 | 5108 | 1296 | 2.842123 | 0.002 | |
| 8010..8650 | 8199 | 641 | 1.73757 | 0.04 | |
| 12670..14150 | 13180 | 1481 | 1.501619 | 0.06 | |
| 16431..17885 | 17376 | 1455 | 1.87441 | 0.03 | |
| 19192..20698 | 19686 | 1507 | 1.331311 | 0.09 | |
| 20707..21358 | 20849 | 652 | 1.830521 | 0.03 | |
| 22749..24029 | 23089 | 1281 | 2.661536 | 0.003 | |
| 24865..26214 | 25262 | 1350 | 2.937666 | 0.001 | |
| 28595..29939 | 29105 | 1345 | 2.309257 | 0.01 | |
| 30811..31694 | 31261 | 884 | 2.11002 | 0.01 | |
| 36124..37549 | 37042 | 1426 | 1.682835 | 0.04 | |
| 38675..39920 | 38939 | 1246 | 1.633201 | 0.05 | |
| 42243..42987 | 42644 | 745 | 1.684027 | 0.04 | |
| 59071..59868 | 59353 | 798 | 4.075761 | 2.29E-05 | |
| 61965..63174 | 62175 | 1210 | 2.59355 | 0.004 | |
| 63545..64951 | 64473 | 1407 | 1.445806 | 0.07 | |
| 65799..67146 | 66233 | 1348 | 1.360798 | 0.08 | |
| 68405..69276 | 68787 | 872 | 2.694848 | 0.003 | |
| 70267..70901 | 70726 | 635 | 1.514277 | 0.06 | |
| 72045..72723 | 72389 | 679 | 1.518148 | 0.06 | |
| 74933..76278 | 75279 | 1346 | 2.194771 | 0.01 | |
| 76476..77548 | 77050 | 1073 | 1.302639 | 0.09 | |
| 81098..81969 | 81394 | 872 | 1.476523 | 0.06 | |
| 85641..86767 | 86030 | 1127 | 2.093028 | 0.01 | |
| 90430..91540 | 91373 | 1111 | 2.710086 | 0.003 | |
| 93302..94678 | 94284 | 1377 | 2.754206 | 0.002 | |
| 98460..99627 | 98968 | 1168 | 1.42698 | 0.07 | |
| 114698..116168 | 115201 | 1471 | 1.968998 | 0.02 | |
| 117026..118130 | 117731 | 1105 | 2.104799 | 0.01 | |
| 119803..120219 | 120101 | 417 | 1.943905 | 0.02 | |
| 121704..122947 | 122626 | 1244 | 2.074034 | 0.01 | |
| 124230..125479 | 124471 | 1250 | 5.775044 | 3.85E-09 | |
| 126352..127174 | 126695 | 823 | 6.107458 | 5.06E-10 | |
| 127960..128685 | 128182 | 726 | 1.554884 | 0.05 | |
| 129178..130621 | 130141 | 1444 | 2.674637 | 0.003 | |
| 135623..136486 | 135950 | 864 | 4.658055 | 1.6E-06 | |
| 136938..137797 | 137097 | 860 | 4.912278 | 4.5E-07 | |
